# Relevance of Protein–Polysaccharide Interactions on Nutritional Quality and Gastrointestinal Digestion of Protein-Based Foods

**DOI:** 10.1021/acs.jafc.4c11949

**Published:** 2025-02-18

**Authors:** Cynthia Fontes-Candia, Laura Díaz-Piñero, Laura María Vega-Gómez, Irene Molina-Gilarranz, Marta Martínez-Sanz

**Affiliations:** † Department of Food Chemistry and Technology, 34344 Teagasc Food Research Centre, County Cork, Fermoy P61 C996, Ireland; ‡ Instituto de Investigación en Ciencias de la Alimentación, 370420 CIAL (CSIC−UAM, CEI UAM + CSIC), Calle Nicolás Cabrera 9, 28049 Madrid, Spain; § Escuela de Doctorado, Universidad Autónoma de Madrid, Francisco Tomas y Valiente 7, 28049 Madrid, Spain; ∥ Departamento de Química Agrícola y Bromatología, Science Faculty, Universidad Autónoma de Madrid, Francisco Tomas y Valiente 7, 28049 Madrid, Spain

**Keywords:** alternative proteins, digestibility, techno-functional properties, fibers

## Abstract

Protein–polysaccharide interactions are key to determine the techno-functional and nutritional properties of food systems. Proteins and polysaccharides can form complexes that strongly affect the digestion mechanism by different pathways. Polysaccharides may reduce protein digestibility by altering protein conformation, increasing the viscosity of the digestive medium, inhibiting digestive enzymes, and/or promoting or hindering interactions with physiological components, such as bile salts and phospholipids. This is expected to affect the intestinal transport process and bioavailability of nutrients. Thus, understanding the mechanism and impact of protein–polysaccharide interactions is crucial for designing efficient processing strategies and predicting the nutritional impact of foods.

## Graphical abstract

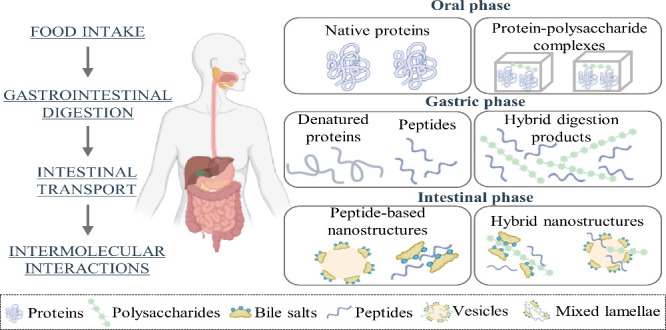

## 1 Introduction

Proteins and polysaccharides are two of the most important macronutrients present in foods. They are widely used as functional ingredients in many food products mainly due to their ability to improve some techno-functional properties, such as mechanical performance, rheology, and stability.^1^ In particular, polysaccharides are hydrophilic biopolymers, which are commonly used in the food industry as thickening, gelling, and stabilizing agents. On the other hand, proteins are used as stabilizing agents, being more commonly used in emulsion systems.^2^ Because both components are typically found in many food products (either naturally or as additives), it is essential to understand how the interactions that can be potentially established between them may affect the nutritional and techno-functional properties of food. This is particularly relevant in the food industry, where consumer demand drives the development of nutritionally enhanced, clean-label products while promoting plant-based protein sources to mitigate the environmental and health impacts of excessive animal resource consumption.

Polysaccharide–protein interactions take place in many different types of foods; however, they are particularly relevant in the case of plants, as they are key to their structural components, i.e., the cell walls.^3^ Plant cell walls are composed of a complex network of fibrillar, skeletal, or matrix polysaccharides, proteins, and aliphatic aromatic compounds.^4^ The structure of their individual components has been extensively studied; however, the interaction mechanism between them and their spatial disposition is still under investigation. Apart from their obvious biological relevance, these interactions in natural food sources are key to determine their techno-functional properties, nutritional quality, and behavior upon processing. Understanding these interactions, influenced by factors like pH, temperature, and processing methods, is vital for designing efficient processing strategies and predicting the nutritional impact of such foods.^5^

## 2 Exploiting Protein–Polysaccharide Interactions for the Design of Novel Food Products and Nutraceuticals

Protein–polysaccharide interactions can also be deliberately manipulated to modify the techno-functional and nutritional properties of foods and ingredients. This can be particularly interesting for the development of foods with specific rheological or textural properties, functional foods with increased satiating effect, bioactive ingredients with controlled release of active components, and stabilized emulsion systems with a limited rate of lipolysis during gastrointestinal digestion.^6^ These complexes can form gel-like structures with a broad range of rheological properties, which can be tuned by selecting the proper blends of polysaccharides and proteins.^7^ When proteins are mixed with polysaccharides in aqueous solutions, three distinct situations can occur: (i) phase separation (thermodynamic incompatibility), (ii) formation of a homogeneous solution, where both components do not interact (co-solubility or miscibility), and (iii) formation of a two-phase system, with both components in the same phase (complexation or co-acervation).^3,8^ Protein–polysaccharide complexes can be formed by covalent bonds (Maillard conjugates) or non-covalent bonds (hydrogen bond, electrostatic attraction, and hydrophobic interactions).^1^ Apart from the molecular structure of each component, different parameters such as ionic strength, pH, concentration, and temperature, among others, may determine the type of interactions established. However, it should be noted that, most frequently, different types of interactions co-exist.

Proteins and polysaccharides exhibit charge-based electrostatic interactions, often influenced by the ionic strength. In proteins, ionic strength can induce conformational changes, affecting binding affinity by attenuating charges, reducing repulsive forces, and promoting particle aggregation.^9^ For polysaccharides, the ionic strength impacts their conformation (coil or uncoil), altering their interaction with proteins.

In addition, pH plays a very important role in the solubility and surface charges of proteins and polysaccharides, affecting their binding capacity and overall behavior, as schematically shown in Figure 1. In particular, the pH affects the degree of dissociation of the charged groups in the formed complexes, thus changing their positive or negative charges.^9^ For proteins, pH below the isoelectric point (pI) results in positively charged amino groups, while, above the pI, carboxyl groups become negatively charged, potentially exposing or hiding binding sites. At the same time, it should be considered that proteins present less solubility near the isoelectric point. On the other hand, polysaccharides with ionizable groups, such as carboxyl or sulfate, also undergo pH-dependent conformational changes, impacting gelling properties and protein interactions. Thus, negatively charged proteins and polysaccharides can form stable dispersions due to their electrostatic repulsion. On the other hand, oppositely charged proteins and polysaccharides can produce complexes by electrostatic interactions.^1^

**Figure 1:**
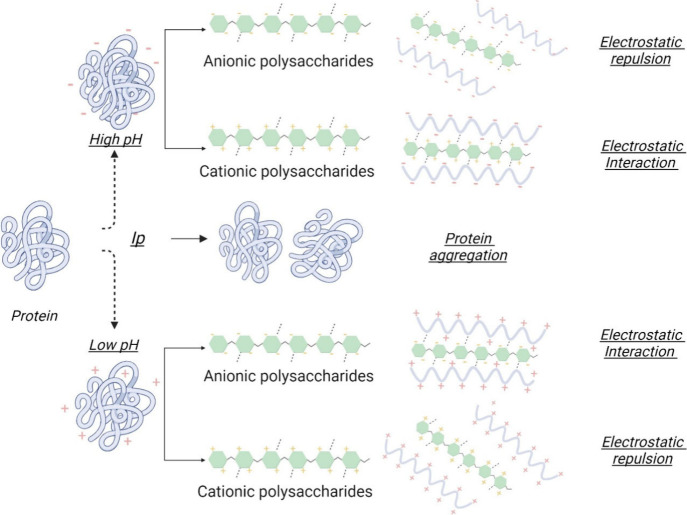
Effect of pH on protein–polysaccharide interactions.

The temperature also plays a very important role in the interactions between polysaccharides and proteins as hydrogen-bonding and hydrophobic interactions are temperature-dependent,^5^ and therefore, thermal processing will have an impact on them. In addition, the conformation of proteins can be strongly affected by the temperature as, if they are subjected to relatively high temperatures, they may denature, adopting unfolded conformations, which allow for greater interactions by having more reactive sites exposed.^2^

In general, the gelling capacity of polysaccharides can be exploited to produce protein-rich alternative products, such as vegan “gelatin”, while their thickening ability can be used to generate products with paste-like consistency or to improve the texture, as in dairy products.^10^ Furthermore, adding polysaccharides to protein-stabilized emulsions or foams can increase their stability via the formation of protein–polysaccharide complexes at the oil/water interface.^1^ Another interesting application is the development of controlled delivery systems in bioactive food products and nutraceuticals.^11^ These systems include emulsion- and capsule-based delivery systems, nanogels, composite nanoparticles, molecular complexes, core–shell particles, and micelles.^12^ These delivery systems offer a physical barrier against environmental stresses, to protect labile bioactive molecules, improving bioaccessibility of some compounds, which can enhance their cellular uptake.^12^ In some cases, polysaccharides are used to encapsulate proteins, reducing proteolysis in the stomach and promoting satiety.^13,14^ In that case, the type of polysaccharide used, the polysaccharide/protein ratio, and the environmental pH are relevant factors to consider in the design of these systems,^15^ where the structure also influences their functional and nutritional properties.^14^

## 3 Gastrointestinal Digestion of Protein–Polysaccharide Systems

During gastrointestinal digestion, food experiences physical and chemical transformations facilitated by chewing, peristalsis, pH levels, and digestive enzymes. Such a complex process needs to be studied individually for each food product, because compositional or structural changes in their formulation can lead to major effects on the digestion mechanism. In response to the substantial expansion of the food industry, with new ingredients or products being continuously generated, and due to the impracticality and negative ethical implications of testing all of these new products through *in vivo* experiments, involving the use of animals or humans, *in vitro* digestion methods have arisen as a sustainable alternative. Over the years, various protocols have emerged, with the INFOGEST standardized method being the most commonly used, due to its strong correlation with *in vivo* data.^16,17^

To date, the digestibility of proteins and polysaccharides has been studied separately, and the effect of the combination of both components in foods is still being investigated. The thousands of potential variations of the structure of both biomolecules individually and their multiple types of interactions make this a very complex matter of study. As summarized in Figure 2, protein–polysaccharide interactions may have a strong impact on the digestion process due to different aspects, such as reducing enzyme effectiveness, interacting with various components of the digestive medium (i.e., salts and phospholipids), modifying the viscosity of the medium, and promoting or hindering interactions with components of the digestive medium.

**Figure 2:**
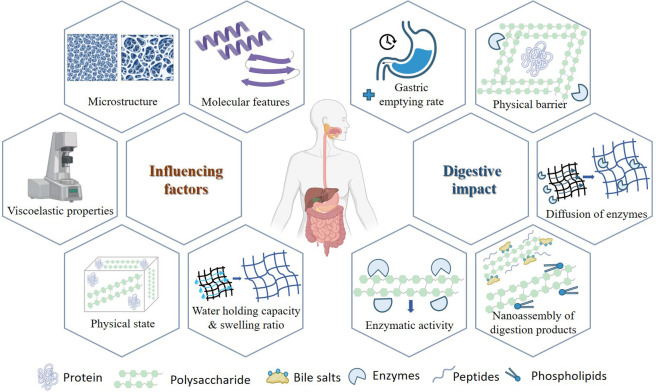
Factors influencing protein–polysaccharide interactions and their effect on the gastrointestinal digestion mechanism.

### 3.1 Effect of the Physicochemical Features of Protein–Polysaccharide Systems

The addition of polysaccharides to a protein system induces structural modifications in the three-dimensional network forming mixed gelling systems.^18^ In some cases, polysaccharides cause proteins to unfold, altering both the secondary and tertiary structures of proteins, promoting the transition from α-helix to β-sheet structures, and shifting the all-*gauche* conformation to *trans*–*gauche*–*gauche*.^19^ This transition enhances the water-holding capacity (WHC) of protein gels due to the abundance of hydrophilic groups, which promote hydrogen bonding with the protein’s amide, carboxyl, amino, and hydroxyl groups, and reduces water mobility.^20^ These changes on WHC will influence digestion, by increasing gel stiffness as well as affecting the microstructure and swelling ratio.^18^ For instance, a significant negative correlation was found between gel hardness and the digestion rate being the gel microstructure strongly influenced by the surface charge of carboxymethylated cellulose nanofibrils, with a denser microstructure potentially delaying the gastric digestibility of myofibrillar protein gels.^21^

In the case of polysaccharide–protein gel-like systems, the microstructure (porosity and pore size) is largely influenced by the gelation mechanism. This will ultimately affect the gel strength and WHC.^7^ A less porous structure is closely linked to increased hardness, network uniformity, and cohesiveness. In contrast, a more elastic texture is associated with larger pores. Thus, the more porous structures lead to a higher diffusion and swelling ratio of the digestion medium, increasing the proteolytic effect.^19^ The swelling properties will also be governed by the degree of ionization, electrostatic interactions, and balance between hydrophilic and hydrophobic forces, with the swelling ratio being directly linked to structural erosion and degradation, which, in turn, leads to an increased digestion rate.^19^ For example, research on myofibrillar proteins revealed a significant correlation between the diffusion rate of pepsin and the degree of hydrolysis. This work suggested that water mobility may influence pepsin diffusion, thereby influencing the gastric digestion of myofibrillar protein gels.^21^ Moreover, some studies have reported that the interaction between the carboxylic and amino groups in the peptide chains of proteins and the negatively charged groups on polysaccharides hinder protein hydrolysis, hence reducing protein digestibility.^22^ This was reported in a study where different polysaccharides (carrageenan, gum arabic, locust bean gum, alginic acid, and citrus pectin) showed an impact on casein digestibility, mainly attributed to interactions between fibers and either the enzymes or casein rather than changes in the viscosity of the mixtures.^23^

### 3.2 Effect of Protein–Polysaccharide Interactions on the Digestion Kinetics

The digestion of proteins was influenced by factors such as the protein structure, folding interactions, and residue hydrophobicity, significantly affecting proteolysis. It is important to consider that even minor conformational changes can drastically alter protein digestibility. Process-induced changes, pH, and the presence of functional groups must be carefully considered, as they can impact protein conformation and overall digestibility.^24^

In contrast, for polysaccharides, it is important to note that human digestive enzymes are only able to hydrolyze certain types, such as starch, while others, widely known as dietary fibers, largely resist digestion. Among dietary fibers, we find soluble (e.g., agar and pectin) and insoluble (e.g., cellulose) fibers, which will behave differently during the digestion. Dietary fibers have the capacity to absorb a large amount of water, which subsequently expands in the stomach to further increase the sense of satiety. This will affect the viscosity of the food bolus, which may slow stomach emptying and prolong the feeling of fullness.^25^ Thus, the presence of these polysaccharides in natural food substrates and the presence of polysaccharides as additives (e.g., thickeners) in food products are expected to have a significant impact on the overall digestion process. This effect slows molecular movement and creates a denser, more compact structure, which limits access to digestive enzymes and gastric juices.^26^

Protein–polysaccharide interactions play a significant role in modulating digestion, as their association with polysaccharides has been shown to extend digestion time by slowing stomach emptying.^27^ This effect has prompted extensive investigation into the *in vitro* gastrointestinal digestion of protein–polysaccharide complexes. Several studies have revealed the importance of rheological properties on the digestion process, while other studies have found that structural characteristics are of greater relevance. For example, xanthan gum and carrageenan were digested in combination with soybean protein isolate (SPI), observing that SPI digestibility was delayed due to the presence of the polysaccharides, with this effect being more evident with the more negatively charged polysaccharides.^27^ Gel strength varied with the type of polysaccharide, forming stronger gels with carrageenan. This was also an important factor affecting the digestion process.^13,14,28^

Polysaccharides also play a multifaceted role in influencing protein digestibility within hybrid protein–polysaccharide hydrogels by acting as a physical barrier and reducing enzyme penetration into the gel structure, thereby slowing protein degradation.^14,20^ This phenomenon was observed in soybean curd during *in vitro* digestion, where reduced diffusion of enzymes, caused by microstructural changes, limited the contact surface area between proteins and enzymes. The formation of hydrogen bonds and cross-links further obstructed enzyme access to protein binding sites, emphasizing the impact of polysaccharides on protein hydrolysis and digestion efficiency. Therefore, the fiber source as well as the physical state of the food and cell wall layers must be considered when evaluating variations in protein digestibility.^20^

In addition to acting as a physical barrier, polysaccharides can influence proteolysis in other ways. Reduced digestibility may also result from the negative impact of certain polysaccharides on digestive enzyme activity. This reduction in enzyme activity can be attributed to the non-specific enzyme binding or, in the case of non-purified fiber sources, the presence of specific enzyme inhibitors.^29^ Such inhibition can affect both proteases and other digestive enzymes, as reported for α-amylase activity, which influences glucose metabolism.^22^ Despite the fact that different fibers might interact with digestive enzymes and alter their activity, according to *in vitro* studies, they do not decrease the overall measurable activity of digestive enzymes in the gut contents.^30^

Dietary fibers also exhibit the capacity to bind with bile acids, with this capacity being variable depending upon the type of fibers and their structure.^31,32^ In addition, cross-linking of protein–polysaccharide hybrid gels has been reported to promote the binding of bile acids.^33^ Moreover, dietary fibers could also potentially bind to phospholipids, found in mixed micelles along with bile salts, increasing their fecal excretion.^34^ However, the pattern of phospholipid binding shows some variation compared to bile acids.^35^ This interaction of polysaccharides with bile salts and phospholipids in the small intestine may affect lipid digestion by decreasing the availability of surface-active components needed to stabilize lipid droplets (triacylglycerols) or to facilitate the transport of lipid digestion products (free fatty acids and monoacylglycerols) from the droplet surfaces to the intestine epithelial cells.^36^ In addition to their effect during proteolysis along the digestive tract, it has been observed that certain polysaccharides can influence peptide absorption by increasing intestinal permeability, thereby enhancing the bioavailability of proteins.^37,38^

Similarly, the digestion of polysaccharides can be influenced by the presence of proteins. This was demonstrated in a study in which wheat flour, composed of a matrix of starch and proteins, was digested. Results showed that a higher protein content led to a higher resistance to digestion by α-amylase.^39^ Thus, protein–polysaccharide interactions will not only have an impact on the digestion mechanism and hydrolysis rate, but they are also expected to affect the nanostructural assembly of the digestion products generated.^40^ This is crucial for nutrient assimilation and the signaling effects of certain digestion products on enteroendocrine cells. While the relationship between digestion product nanostructures and their intestinal transport or signaling remains poorly understood, advancements in structural characterization tools are expected to provide valuable insights.

### 3.3 Further Implications on Metabolic Responses

The types of structures formed during digestion and the mechanisms of intestinal transport are particularly important to determine their impact on potential metabolic responses induced by food digestion products. Although obviously more limited in number, due to their complexity and ethical implications, some *in vivo* digestion studies have also been conducted in polysaccharide/protein systems to investigate the effect of the protein–polysaccharide interaction on the metabolic responses. In this case, studies are mostly focused on delivery systems rather than food products. As an example, rice selenium-containing peptide TSeMMM was encapsulated within zein and gum arabic, and the obtained nanoparticles were administered to mice for 4 h in different concentrations. The results of this animal trial showed that the nanoparticles enhanced the oral bioavailability of peptides and affected tissue glutathione levels, proving the positive effect of protein–polysaccharide interactions as encapsulation systems.^41^ Wang et al. reported that protein-enriched fiber meals prevent weight gain by promoting satiety and reducing the food intake in rats, thus demonstrating a synergic effect of both components.^25^

The impact of viscous soluble dietary fibers on energy regulation has also been explored. Researchers found a reduction in the apparent protein digestibility, which could be attributed to two main factors. First, dietary fibers sterically hinder the interaction between carbohydrates and digestive enzymes, reducing both enzyme diffusion and enzyme–substrate encounters, which, in turn, slows nutrient digestion and absorption. Second, the high viscosity slows digestion, prolongs gastric emptying, and extends the time nutrients stay in the intestine, leading to a more gradual release of glucose into the bloodstream.^42^ These findings suggest that soluble dietary fibers with high viscosity could be effective ingredients in foods designed to mitigate postprandial glucose spikes in humans.^42^ Furthermore, some studies have observed the inhibition of digestive enzymes along with compensatory increases in pancreatic–biliary secretions in alginate-fed rats. These findings support the idea that alginates disrupt digestive processes, slowing nutrient absorption and reducing postprandial glucose spikes. Additionally, the increase in bowel mass reported in several rodent studies is considered an adaptive response to decreased nutrient digestibility.^43^

## 4 Future Perspectives

Investigation of protein–polysaccharide interactions is highly relevant to transform the food industry in the current context of climate change, profound socioeconomic changes, and global health issues. This knowledge will be crucial for the design of novel food products based on alternative protein sources with improved nutritional properties and produced sustainably.

The study of protein–polysaccharide interactions is expected to bring relevant insights in the following years for the exploitation of alternative protein sources. In particular, studying the role of these interactions on the structure of cell walls will help to evaluate the digestibility and bioavailability of nutrients in these sources as well as to design efficient processing methods to enhance their nutritional and techno-functional properties. Furthermore, some works have already explored the possibility of deliberately exploiting these interactions to generate hybrid structures to trigger specific metabolic responses (e.g., increased satiety, reduced blood cholesterol, etc.).

Despite this, knowledge of the impact of protein–polysaccharide interactions during the gastrointestinal digestion process still remains limited. The interactions established upon hydrolysis of food components and components of the physiological medium, the types of nanostructures formed as a result, and the mechanisms of intestinal transport are particularly important to determine the impact on potential metabolic responses induced by food products. Addressing these complex questions will require a comprehensive and multidisciplinary approach. This can be achieved by combining advanced characterization methods, such as rheology, scattering techniques, and peptidomics, to elucidate the structure at multiple length scales, together with biotechnology approaches, to understand how nanostructure and intestinal transport processes are correlated. Only in this way will it be possible to conduct a more rational and efficient design of novel food products for the future.
